# Influenza virus infections in patients with malignancies –– characteristics and outcome of the season 2014/15. A survey conducted by the Infectious Diseases Working Party (AGIHO) of the German Society of Haematology and Medical Oncology (DGHO)

**DOI:** 10.1007/s10096-016-2833-3

**Published:** 2016-11-12

**Authors:** B. Hermann, N. Lehners, M. Brodhun, K. Boden, A. Hochhaus, M. Kochanek, K. Meckel, K. Mayer, T. Rachow, C. Rieger, E. Schalk, T. Weber, A. Schmeier-Jürchott, P. Schlattmann, D. Teschner, M. von Lilienfeld-Toal

**Affiliations:** 1 Leibniz Institut für Naturstoff-Forschung und Infektionsbiologie, Hans-Knöll-Institut, 07745 Jena, Germany; 2 Department of Haematology and Oncology, University Hospital Heidelberg, Heidelberg, Germany; 3 Medizinische Klinik II, Abteilung für Haematologie und internistische Onkologie, Universitätsklinikum Jena, Jena, Germany; 4 Institut für Klinische Chemie und Laboratoriumsmedizin, University Hospital Jena, Jena, Germany; 5 Department I of Internal Medicine, University Hospital of Cologne, Cologne, Germany; 6 Medizinische Klinik III, University Hospital Bonn, Bonn, Germany; 7 Internistische Lehrpraxis der Ludwig-Maximilians-Universität München, University of Munich, Munich, Germany; 8 Otto-von-Guericke University Magdeburg, Medical Centre, Department of Haematology and Oncology, Magdeburg, Germany; 9 University Hospital Halle, Halle, Germany; 10 University Medical Centre of the Johannes Gutenberg University, Mainz, Germany; 11 Department of Medical Statistics, Informatics and Documentation, University Hospital Jena, Jena, Germany; 12 Forschungscampus InfectoGnostics, Jena, Germany; 13 Integriertes Forschungs- und Behandlungszentrum Sepsis und Sepsisfolgen (CSCC), Universitätsklinikum Jena, Jena, Germany

## Abstract

Influenza virus infections (IVI) may pose a vital threat to immunocompromised patients such as those suffering from malignancies, but specific data on epidemiology and outcome in these patients are scarce. In this study, we collected data on patients with active cancer or with a history of cancer, presenting with documented IVI in eight centres in Germany. Two hundred and three patients were identified, suffering from haematological malignancies or solid tumours; 109 (54 %) patients had active malignant disease. Influenza A was detected in 155 (77 %) and Influenza B in 46 (23 %) of patients (genera not determined in two patients). Clinical symptoms were consistent with upper respiratory tract infection in 55/203 (27 %), influenza-like illness in 82/203 (40 %), and pneumonia in 67/203 (33 %). Anti-viral treatment with oseltamivir was received by 116/195 (59 %). Superinfections occurred in 37/203 (18 %), and admission on an intensive care unit was required in 26/203 (13 %). Seventeen patients (9 %) died. Independent risk factors for death were delayed diagnosis of IVI and bacterial or fungal superinfection, but not underlying malignancy or ongoing immunosuppression. In conclusion, patients with IVI show high rates of pneumonia and mortality. Early and rapid diagnosis is essential. The high rate of pneumonia and superinfections should be taken into account when managing IVI in these patients.

## Introduction

Infections are the main cause of treatment-related mortality in cancer patients. Whereas bacterial and fungal infections are common and well-known, infections by community acquired respiratory viruses have often received less attention in the past. With the emergence of nucleic acid amplification techniques (NAT), diagnosis of specific viral infections has become easier and faster, making it possible to gain more precise information on epidemiology and outcome. Most studies with regard to infections with community acquired respiratory viruses, including influenza, in the immunocompromised host have been performed in patients with haematological malignancies, especially those following stem-cell transplantation (SCT) [1–7]. However, there is an evident need to obtain information on the impact of IVI in patients with all kinds of malignancies, as only a few studies have dealt with patients suffering from solid tumours [8]. In the 2014/15 season, the wave of IVI was particularly strong in Germany [9]. It started in October 2014 from and affecting especially the south-east of Germany spreading from there north-west over Germany, peaked in the second week of 2015, and lasted till mid-April 2015 [9]. A genetic drift occurred in the dominating influenza A (H3N2) viruses (62 %), which was not covered by the seasonal vaccine. Influenza A(H1N1)pdm09-viruses were identified in 15 % and influenza B viruses in 23 % of the samples tested positive for influenza [9].

The aim of our study was to understand the clinical epidemiology and outcome of IVI in cancer patients during the 2014/15 influenza season, in order to identify patients at risk of a severe course of infection and mortality.

## Patients and methods

All patients (out- and inpatients) with active malignancy or history of malignant disease presenting with documented IVI to one of the eight participating centres of the AGIHO (at the German university hospitals of Bonn, Cologne, Halle/Saale, Heidelberg, Jena, Magdeburg, Mainz, and Munich) between November 2014 and June 2015 were included in this analysis.

IVI diagnosis was confirmed by tests using nucleic acid amplification techniques (NAT) from respiratory samples. Materials used for NAT analysis were mostly pharyngeal swabs (almost 70 %), but pharyngeal lavage fluids or bronchoalveolar lavage (BAL) fluids were also used. Further influenza A virus subtyping was performed if available. Most centres also tested for respiratory syncytial virus ( RSV), whereas the panel for other respiratory viruses including parainfluenza and rhinoviruses varied according to the different laboratories, and was not prespecified.

Data were collected on each site from medical records using a predefined short questionnaire. The questionnaire included data on demographics (age, sex), type of influenza virus, clinical presentation of infection, treatment and outcome, type and treatment of underlying malignant disease, and immunosuppressive therapy, as well as presence of co-infections. Because data on vaccination status were not available from medical records in most cases, we tried to obtain additional information on this point by (telephone) interview or using the influenza registry of the health authorities in Jena, Heidelberg, and Mainz. Patients were followed with regard to outcome until clinical resolution of the infection, discharge from hospital, or death. Data were transferred to the coordinating centre (Jena) in an anonymised form.

Influenza-like illness (ILI) is defined according to the European Centre for Disease Prevention and Control (ECDC) by at least one of four systemic symptoms (fever, malaise, headache, or myalgia) and at least one respiratory symptom (cough, sore throat, or shortness of breath). A sudden onset of symptoms is mandatory [10].

Upper respiratory tract infection (URTI) is anatomically defined to include sinusitis and rhinitis and is characterized by new onset of symptoms including at least one of cough, coryza, sore throat, or shortness of breath.

Lower respiratory tract infection (LRTI) involving the pulmonary parenchyma, is defined by clinical or radiological evidence of pneumonia [11]. Severe LRTI was defined as requirement of treatment on intensive care unit (ICU) or death.

Detection of bacterial or fungal pathogens in BAL or blood culture (BC) was considered a relevant superinfection except for the following conditions: In the case of coagulase-negative staphylococci, the detection of the pathogen in at least two different BC was required for definition as relevant superinfection. *Enterococcus spp*. were considered relevant if detected in BC but not if derived from respiratory samples. *Escherichia coli*, *Pseudomonas aeruginosa*, and *Stenotrophomonas maltophilia* cultured from pharyngeal lavage fluids were considered contaminants. Detection of respiratory viruses in samples derived from the respiratory tract was considered a relevant co-infection, whereas detection of herpes viruses was classified as endogenous reactivation secondary to IVI. For statistical analysis, all types of relevant infection were summarized and classified as superinfection.

Statistical analysis was performed with IBM SPSS statistics software, version 21. Proportions were analysed using chi-square tests. Time to event data were analysed using the Kaplan–Meier method and the log-rank for univariable analyses. Multivariable analyses are based on Cox regression models. A two-sided *p*-value <0.05 was considered significant.

## Results

A cohort of 210 cancer patients presenting with IVI was identified. Of those, complete clinical information was available in 203 patients who were included in the final analysis. The median follow- up interval was 127 days (range 0–236 days). The median age was 61 years (interquartile range [IQR] 49–65 years) and 61 % of the patients were male (123/203, Table 1). The majority of patients (158/203, 78 %) suffered from haematological malignancies, and most (106/203, 52 %) had received any type of SCT. There was also a considerable number of patients with solid tumours (21/203, 10 %). About half of the patients (109/203, 54 %) had active malignant disease at the time of IVI diagnosis (Table 1).

**Table 1:** Patient characteristics

	All	Myeloid malignancies^a^	Lymphoid malignancies^b^	Solid tumours ^c^	Others^g^	*P* value^d^
	*N* = 203	*N* = 51	*N* = 107	*N* = 21	*N* = 24	
Age (median, IQR)	61 (49–65)	57 (48–62)	61 (49–70)	63 (61–73)	62 (47–64)	0.019
Male: *n*/*N* (%)	123/203 (61 %)	26/51 (51 %)	69/107 (65 %)	14/21 (67 %)	14/24 (58 %)	0.39
Active malignant disease: *n*/*N* (%)	109/200 (55 %)	15/49 (31 %)	69/107 (65 %)	15/21 (71 %)	10/23 (44 %)	<0.001
Stem cell transplantation: *n*/*N* (%)	105/202 (52 %)					<0.001
- Autologous	28/202 (14 %)	0/51	28/106 (26 %)	0/21	0/24	
- Allogeneic	77/202 (38 %)	37/51 (73 %)	28/106 (26 %)	0/21	12/24 (50 %)	
Type of influenza: *n*/*N* (%)						0.33
- A	155/201 (77 %)	41/51 (80 %)	80/107 (75 %)	15/21 (71 %)	19/24 (79 %)	
- B	46/201 (23 %)	10/51 (20 %)	25/107 (23 %)	6/21 (29 %)	5/24 (21 %)	
- Not specified	2/201 (1 %)	0/51	2/107 (2 %)	0/21		
Sample collected: *n*/*N* (%)						0.07
- Swab	139/203 (69 %)	33/51 (65 %)	75/107 (70 %)	9/21 (43 %)	22/24 (92 %)	
- Pharyngeal lavage fluid	42/203 (21 %)	11/51 (22 %)	22/107 (21 %)	7/21 (33 %)	2/24 (8 %)	
- BAL	19/203 (9 %)	6/51 (12 %)	8/107 (8 %)	5/21 (24 %)	0/24	
- Other	3/203 (1 %)	1/52 (2 %)	2/107 (2 %)	0/21	0/24	
Initial symptoms: *n*/*N* (%)						0.005
- Asymptomatic	9/203 (4 %)	0/51	7/107 (7 %)	1/21 (5 %)	1/24 (4 %)	
- URTI	55/203 (26 %)	15/51 (29 %)	19/107 (18 %)	7/21 (33 %)	14/24 (58 %)	
- ILI	82/203 (39 %)	25/51 (49 %)	47/107 (44 %)	7/21 (33 %)	3/24 (13 %)	
- Pneumonia	57/203^f^ (27 %)	11/51 (22 %)	34/107 (32 %)	6/21 (29 %)	6/24 (25 %)	
ICU: *n*/*N* (%)	26/201 (13 %)	6/50 (12 %)	17/107 (16 %)	3/21 (15 %)	0/24	0.2
Death from influenza: *n*/*N* (%)	17/200 (9 %)	5/50 (10 %)	10/105 (10 %)	1/21 (4 %)	1/24 (4 %)	0.7
Treatment with oseltamivir: *n*/*N* (%)	116/195 (60 %)	31/49 (63 %)	59/102 (58 %)	14/21 (67 %)	12/23 (52 %)	0.7
Superinfection^e:^: *n*/*N* (%)	37/201 (18 %)	12/51 (24 %)	21/106 (20 %)	6/20 (30 %)	3/24 (13 %)	0.73
- Bacterial	14	5	7	2	0	
- Fungal	10	2	6	2	0	
- Viral	18	5	8	2	3	

^a^ acute myeloblastic leukaemia (AML) = 43, chronic myeloid leukaemia (CML) = 3, myelodysplastic syndrome (MDS) = 5

^b^ acute lymphoblastic leukaemia (ALL) =13, Hodgkin’s lymphoma =11, Non Hodgkin’s lymphoma (NHL) =36, multiple myeloma (MM) = 47

^c^ gastrointestinal tumours = 4, lung cancer = 9, gynaecological cancer = 3, primitive neuroectodermal tumour (PNET) = 1, sarcoma = 1, urothel carcinoma = 1, melanoma = 1, larynx carcinoma = 1

^d^ Chi-square test

^e^ including co- infections and viral reactivation

^f^ ten additional patients developed pneumonia later on.

^g^ underlying malignancy not specified

Initial clinical presentation included symptoms related to URTI in 55 patients (27 %), ILI in 82 patients (40 %), and pneumonia in 57 patients (28 %). Another ten patients (5 %) developed pneumonia later on, to account for an overall rate of pneumonia of 33 %. Unexpectedly, 21 patients (10 %) also complained of gastrointestinal symptoms such as diarrhoea. Nine patients (4 %) were asymptomatic at the time of diagnosis.

In accordance with the general epidemiology, the dominating influenza virus genus was influenza A (155/201, 77 %), including H3N2 (28 patients) and A(H1N1)pdm09 (six patients) or influenza B (46/201, 23 %; Table 1).

In total, 37 patients (18 %) were considered suffering from relevant superinfection. Two or more different relevant pathogens were detected in samples from seven patients. Ten patients had bacteraemia (one with viral co-infection), and three patients had bacterial pneumonia without positive BC. Another ten patients had fungal pneumonia (one of them with concomitant *E. coli* bacteraemia and one as a double infection with *Aspergillus fumigatus* and *Pneumocystis jirovecii*). The remaining 14 patients had one or more co-infecting viruses. For a list of pathogens causing super- and co-infection, see Table 2. The most frequent pathogens were respiratory viruses, enteric bacteria, and fungi. To a lesser extent, we detected non-fermenting Gram-negative bacteria and Gram-positive bacteria. In four cases we also found reactivation of herpesviridae (HSV, CMV, and HHV6) during IVI, *E. faecium* and *S. maltophilia* in respiratory samples in three cases and in one case respectively, and contamination with coagulase-negative staphylococci in three cases. These findings were considered clinically irrelevant and thus ignored [12].

**Table 2:** Pathogens detected in respiratory samples and blood cultures

Pathogens	Blood	BAL	Sputum/pharyngeal lavage fluid
Viruses			
- RSV			11
- Influenza B virus^$^			1
- Metapneumovirus			1
- Adenovirus			1
- Parainfluenza virus			1
Fungi			
- *Aspergillus spp*.		9	
- *Pneumocystis jirovecii*		1	
- *Scedosporium prolificans*		1	
Gram-positive bacteria			
- *Staphylococcus aureus*			1*
- *Streptococcus pneumoniae*	1		
- *Streptococcus mitis*/*oralis*	1		
- *Enterococcus faecium*	1		
Gram-negative bacteria			
- *Escherichia coli*	3	3	
- *Enterobacter cloacae*	2		
- *Klebsiella pneumoniae*		1	
- *Serratia marcescens*	1	1	
- *Pseudomonas aeruginosa*	3		
- *Stenotrophomonas maltophilia*		1	

BAL = bronchoalveolar lavage fluid

RSV = respiratory syncytial virus

Double and triple infections:

1× Parainfluenza virus and RSV in pharyngeal lavage fluid,

1× Pseudomonas and *E. coli* in BC,

1× *S. mitis* and *E. coli* in BC in addition to *E. coli* in BAL

1× Aspergillus and *E. coli* in BAL,

1× Pseudomonas in BC and adenovirus in sputum,

1× Aspergillus and pneumocystis in BAL

^$^ in a patient with influenza A

* in a patient with pneumonia

One hundred and fourteen patients (56 %) received antiviral treatment. In one case with co-infection with RSV and parainfluenza, ribavirin was administered; all others were treated with oseltamivir. The dose of oseltamivir was 150 mg/d in 89/114 patients (78 %), but some also received a lower dose of 30 to 75 mg/d (14/114, 12 %). The median duration of treatment was 7 days (IQR 6.5–7.5 days).

Information on vaccination against influenza was available in 34 patients only. Twelve of 34 patients were vaccinated in 2014, whereas the other 22 received no vaccination.

Severe course of illness required treatment on intensive care unit (ICU) in 26 cases (13 %). Of 67 patients with LRTI, 23 (34 %) had to be treated on the ICU (nine with bacterial, four with fungal, and two with viral superinfection) and 12 of these patients (18 %) died. Overall, 17/200 patients (9 %) died in the course of IVI (Table 3).

**Table 3:** Characteristics^§^ of deceased patients

Case number	Sex	Age (years)	Malignant disease	Active disease	SCT	Immuno-suppression^a^	Influenza virus genera and subtype	Time to diagnosis ^b^ (days)	Time to death^c^ (days)	Clinical presentation	Oseltamivir	Superinfection	ICU
1	m	48	AML	unknown	allo	unknown	A^+^	6	−2	LRTI	yes	bacterial	yes
2	m	64	MM	no	auto	yes	A^+^	−1	2	LRTI	no	no	no
3	f	66	gynaecological carcinoma	yes	no	no	A H3N2	4	0	LRTI	no	fungal	yes
4	m	52	Hodgkin’s Lymphoma	no	auto	no	A(H1N1)pdm09	7	3	ILI, later LRTI	yes	unknown	yes
5	m	64	AML	no	allo	no	A H3N2	12	17	LRTI	yes	bacterial	yes
6	m	68	NHL	yes	no	yes	B	17	14	LRTI	yes	fungal	yes
7	m	71	MM	yes	auto	yes	A^+^	4	28	LRTI	yes	no	yes
8	f	51	AML	yes	no	yes	A^+^	unknown	9	URTI, later LRTI	yes	viral	yes
9	m	23	ALL	no	allo	no	A^+^	21	11	LRTI	yes	bacterial	yes
10	f	61	MM	yes	auto	yes	A(H1N1)pdm09	8	12	LRTI	yes	fungal	yes
11	f	64	AML	no	allo	no	A H3N2	7	22	LRTI	yes	no	no
12	f	78	MM	yes	no	yes	A^+^	unknown	1	LRTI	unknown	no	no
13	m	68	NHL	no	allo	yes	B	-2	25	ILI, later LRTI	no	no	yes
14	f	85	“other”	yes	no	no	B	21	2	LRTI	no	no	no
15	f	65	AML	no	allo	no	A^+^	3	104*	ILI, later LRTI	yes	bacterial	yes
16	m	77	NHL	yes	no	no	A^+^	15	2	ILI, later LRTI	no	no	no
17	m	65	NHL	yes	yes	no	A H3N2	unknown	87	LRTI	yes	no	yes

^§^Status of vaccination against influenza unknown in all patients

^a^ Any kind of ongoing immunosuppressive therapy

^b^ Duration from start of symptoms to diagnosis influenza

^c^ Duration from diagnosis to death

m = male, f = female,

auto = autologous, allo = allogeneic

ILI = influenza-like illness, LRTI = lower respiratory tract infection, URTI = upper respiratory tract infection

^+^ Influenza A virus not subtyped

*Patient died because of pneumonia, Influenza A was always detected

SCT = stem cell transplantation, AML = acute myeloblastic leukaemia, MM = multiple myeloma, NHL = Non Hodgkin’s lymphoma, ALL = acute lymphoblastic leukaemia

**Fig. 1:**
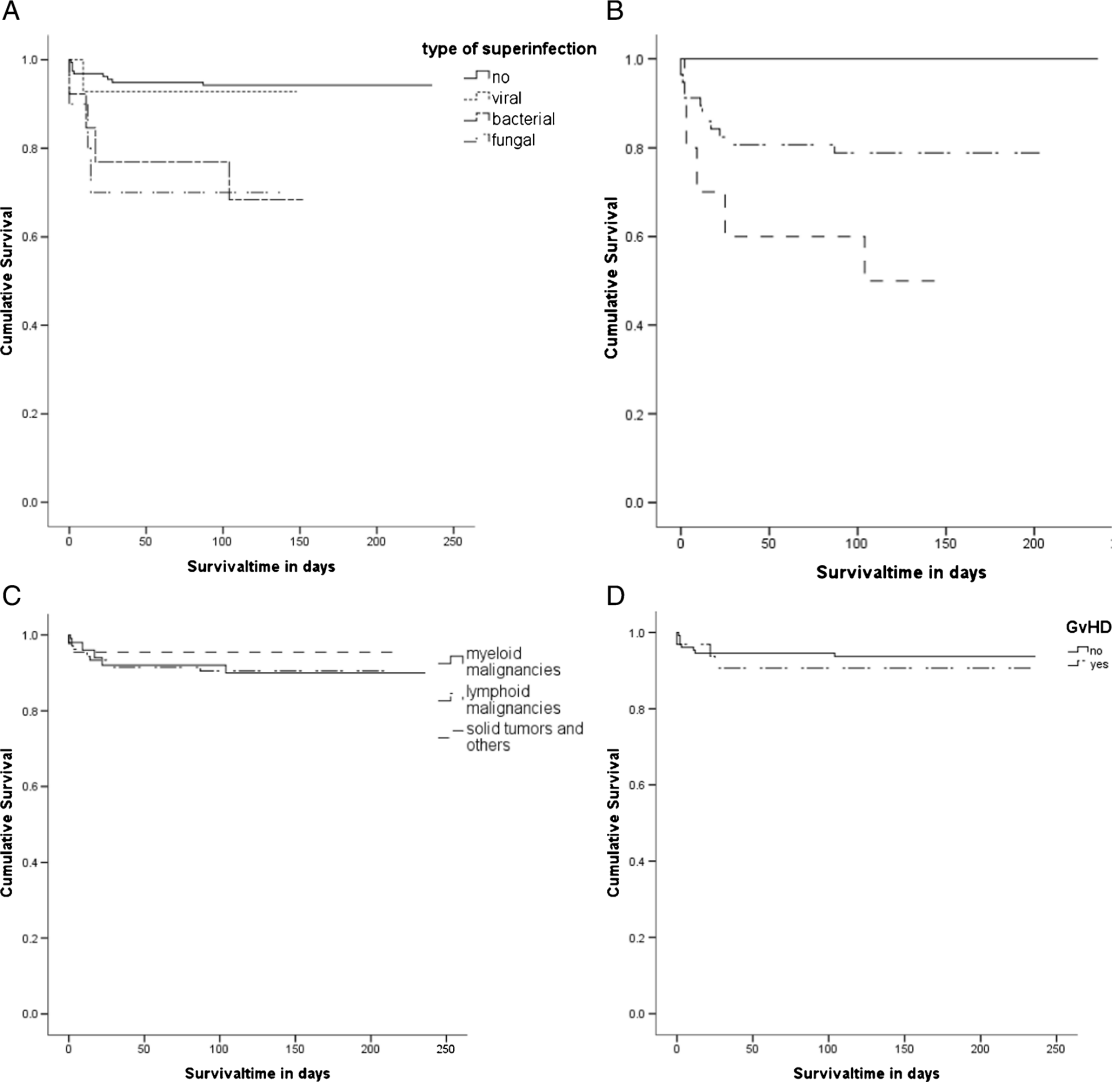
**a** Survival of patients with or without superinfection, *p* = 0.0035 (log-rank). **b** Survival of patients with or without pneumonia, *p* < 0.001 (log-rank). **c** Survival with respect to underlying malignancy, *p* = 0.594 (log-rank). **d** Survival of patients with or without GvHD, *p* = 0.542 (log-rank)

In univariable analysis, prognostic factors for higher mortality were bacterial and fungal superinfection (*p* = 0.0035, Fig. 1a) and presence of pneumonia (*p* < 0.001, Fig. 1b). It should be noted that all patients who died were suffering from LRTI. Furthermore, time from onset of symptoms to diagnosis of IVI was significantly different between survivors and non-survivors (3 days [CI 95 % 2–4 days] versus 7 days [CI 95 % 5–9 days], *p* = 0.002). Patients deceased from influenza-associated causes were older (63 years [IQR 23–85 years]) than patients who survived (57 years [IQR 20–85 years], *p* = 0.043). In contrast, sex, type and activity of malignant disease (Fig. 1c), immunosuppressive therapy, graft versus host disease (GvHD) (Fig. 1d), and treatment with oseltamivir did not significantly influence the outcome in univariable analysis. Likewise, vaccination did not seem to influence mortality significantly (data not shown). Concerning the different viral subtypes, two out of six patients with known A(H1N1)pdm09 infection died, in contrast to four out of 27 with known H3N2 infection (*p* = 0.08).

Multivariable analysis revealed superinfection (hazard ratio [HR] 3.4 [95 % confidence interval (95%CI) 1.09–10.6, *p* = 0.03) and duration from onset of symptoms to diagnosis (HR 1.1 [95%CI 1.01–1.2], *p* = 0.02) as independent prognostic factors.

## Discussion

Due to a high rate of LRTI and a high associated mortality rate, the influenza season 2014/15 represented a relevant threat to cancer patients. At presentation, a third of patients were asymptomatic or presented with URTI only, which was relatively unexpected from a clinician’s point of view. In line with other reports [13], influenza-associated URTI itself was not particularly harmful for the patients as all patients dying from influenza suffered from or developed pneumonia in the course of the illness. However, those patients who developed LRTI during the course of the disease showed a relevant impairment in survival (Fig. 1b), emphasising the need to take IVI seriously regardless of initial presentation.

In our cohort, presence of superinfection and prolonged duration from first symptoms to diagnosis were the sole independent prognostic factors associated with higher mortality. A variety of studies investigating the causes of death in patients with IVI found that notably bacterial and fungal superinfections played an important role with regard to morbidity and mortality [14–16]. However, it seems that the mortality in these patients varies widely between the different cohorts [15], but the reasons for this wide scope are not clear. *Streptococcus pneumoniae*, *Haemophilus influenzae*, *Staphylococcus aureus*, *E. coli*, and *A. fumigatus* are the superinfecting pathogens most frequently described to date [17]. Studies on the pandemics in the twentieth century suggest a high mortality due to superinfections with these pathogens [14]. However, in the era of widespread vaccination against pneumococci in children and the elderly, this might not necessarily be true anymore, as suggested by Tief et al., who reported a low disease severity in children co-infected with influenza and *S. pneumoniae* [18]. In our cohort, 7/23 patients with bacterial or fungal infection died (30 %), showing superinfection to be the strongest predictor of death. Interestingly, we also found *A. fumigatus* to be a relevant pathogen, whereas bacteria typically associated with influenza were outnumbered by enteric bacteria and other Gram-negative bacteria such as *Pseudomonas aeruginosa*. The underrepresentation of Gram-positive bacteria is remarkable and differs from previous reports [15]. The species of bacteria found in our cohort may represent a microbial spectrum originating from the patient’s flora or from the local health care environment. Nosocomial infections like ventilator-associated pneumonia are known to be caused by both Gram-negative organisms such as *Pseudomonas spp*., members of the *Enterobacteriacae*, *Acinetobacter spp*., or *S. maltophilia*, as well as by some Gram-positive organisms such as *S. aureus* [19–22]. Unfortunately, we did not record specifically whether patients were treated as inpatients or outpatients. However, it is not surprising to find a similar spectrum of bacteria in all patients with malignant disease, who, if not hospitalised, usually have close contact to health care institutions. Thus, our findings emphasise that antibiotic therapy for suspected bacterial superinfection in cancer patients with influenza needs to cover Gram-positive as well as Gram-negative bacteria. Moreover, superinfections with *A. fumigatus* are frequent in patients with malignant disease and IVI, highlighting the need for a thorough diagnostic workup and optional antifungal therapy. Screening for viral co-infection was not consistent between the participating centres, which may lead to underestimation of the impact of viral co- infection on the course of IVI in patients with underlying malignant disease.

Time to diagnosis was identified as a second independent prognostic factor for mortality in our study cohort. This may be due to the fact that patients with pneumonia or critically ill patients are usually considered to have other causative pathogens than influenza virus. Therefore, especially at the beginning of a wave of influenza, these patients might be diagnosed too late. Another possible explanation might be that patients with prolonged diagnosis bear the risk of delaying the initiation of treatment with oseltamivir, potentially leading to impaired efficacy of the drug [23]. In our study, time from onset of symptoms to initiation of antiviral treatment was not recorded, therefore we were not able to draw any definite conclusion regarding this point. Nevertheless, our data support the imperative of immediate NAT testing when cancer patients are suspected to suffer from IVI, even at the stage of URTI, to ensure early treatment as recommended [24, 25].

In addition to the microbial and therapy-associated risk factors mentioned above, risk factors for severe influenza in SCT recipients as described by the European Conference on Infections in Leukaemia (ECIL) are: older age, lymphopenia, first 12 months post SCT, GvHD, and immunosuppressive therapy, as well as having an unrelated or mismatched related donor [23]. In our study cohort, we were able to confirm that age has an impact on mortality, whereas GvHD or immunosuppression and prior SCT did not influence the outcome. It is noteworthy that severe disease did not only occur in patients with profound immunosuppression but also in patients with no ongoing active cancer treatment or those with solid tumours, resulting in comparable survival rates (Fig. 1c). This is unexpected, since most reports of life-threatening IVI originate from patients after allogeneic SCT, where substantial immunosuppression is usually an important risk factor [1, 4, 26].

Data on influenza in patients with solid tumours are generally scarce. A large study of 115 patients with solid tumours suffering from influenza found similar results to our cohort of 21 patients with solid tumours: 23 % of these cancer patients presented with pneumonia, and a mortality rate of 10 % was reported [8]. In line with our results, mortality was associated with prolonged duration to diagnosis of IVI. In contrast to our results, ongoing immunosuppression such as cancer treatment was associated with a severe course of viral infection, which was not seen in our patient cohort. Additionally, in this study detailed information on treatment with oseltamivir was provided, showing a benefit for patients being treated early [8]. In our study, oseltamivir did not influence the outcome significantly, possibly due to a delay of therapy.

As we conducted a retrospective study there are several limitations of our analysis due to a lack of data with regard to several issues. Exact information on start of antiviral therapy is lacking as well as the inpatient/outpatient status of the patients. Also, data on vaccination status and subtype of virus are very limited, and we are not able to draw any conclusions regarding the efficacy of vaccination or the virulence of virus subtypes. These questions will have to be addressed in future prospective studies. Nevertheless, all contacts of patients with malignant disease, e.g., partners, household members, and health care workers, should be urged to undergo seasonal influenza vaccination to better protect this vulnerable collective.

## Conclusion

Albeit retrospective, some conclusions can be drawn from our analysis:

Influenza is potentially dangerous due to high rates of pneumonia and high mortality irrespective of the underlying malignant disease and therefore should be taken seriously in all groups of cancer patients. Therefore, an early diagnosis of IVI is imperative. Superinfections need to be addressed immediately and efficiently since this is the most dangerous complication.
